# Development of the Workplace Health Savings Calculator: a practical tool to measure economic impact from reduced absenteeism and staff turnover in workplace health promotion

**DOI:** 10.1186/s13104-015-1402-7

**Published:** 2015-09-18

**Authors:** Siyan Baxter, Sharon Campbell, Kristy Sanderson, Carl Cazaly, Alison Venn, Carole Owen, Andrew J. Palmer

**Affiliations:** Menzies Institute for Medical Research, University of Tasmania, Medical Science 2 Building, 17 Liverpool St, Private Bag 23, Hobart, TAS 7000 Australia; Population Health Services, Department of Health and Human Services (DHHS), 2/25 Argyle St, GPO Box 125, Hobart, TAS 7001 Australia

**Keywords:** Workplace health promotion, Health economics, Return on investment, Calculator, Absenteeism, Staff turnover, Productivity, Workplace, Employee, Policy-research collaboration

## Abstract

**Background:**

Workplace health promotion is focussed on improving the health and wellbeing of workers. Although quantifiable effectiveness and economic evidence is variable, workplace health promotion is recognised by both government and business stakeholders as potentially beneficial for worker health and economic advantage. Despite the current debate on whether conclusive positive outcomes exist, governments are investing, and business engagement is necessary for value to be realised. Practical tools are needed to assist decision makers in developing the business case for workplace health promotion programs. Our primary objective was to develop an evidence-based, simple and easy-to-use resource (calculator) for Australian employers interested in workplace health investment figures.

**Results:**

Three phases were undertaken to develop the calculator. First, evidence from a literature review located appropriate effectiveness measures. Second, a review of employer-facilitated programs aimed at improving the health and wellbeing of employees was utilised to identify change estimates surrounding these measures, and third, currently available online evaluation tools and models were investigated. We present a simple web-based calculator for use by employers who wish to estimate potential annual savings associated with implementing a successful workplace health promotion program. The calculator uses effectiveness measures (absenteeism and staff turnover rates) and change estimates sourced from 55 case studies to generate the annual savings an employer may potentially gain. Australian wage statistics were used to calculate replacement costs due to staff turnover. The calculator was named the Workplace Health Savings Calculator and adapted and reproduced on the Healthy Workers web portal by the Australian Commonwealth Government Department of Health and Ageing.

**Conclusion:**

The Workplace Health Savings Calculator is a simple online business tool that aims to engage employers and to assist participation, development and implementation of workplace health promotion programs.

## Background

Improving the health and wellbeing of workers is firmly on the public health and business agenda. The World Health Organisation (WHO) has identified the workplace as a target setting for health promotion [1], and formed a Global Plan of Action on Workers’ Health (2008–2017) [2] to protect and promote health at work and respond to the health needs of the working population. Endorsement of this action plan is evidenced in the emergent company and society-wide shift to include workplace health promotion as a key strategy. Consequently, workplace health has gained profile as a strategic asset to economies, as revealed in various international reports and policy guidelines [3–9]. This stands, despite recent inconclusive reviews on whether health and economic outcomes are positive, negative or neutral [10–13], and an extensive review that demonstrated economic evidence, although improving over time, is low to moderate in methodological quality [14]. Nonetheless, the evidence that healthy employees provide social and economic benefits to businesses and the community continues to be largely accepted. These include reductions in absenteeism from illness and injury, increased productivity, reduced staff turnover, reduction in health care costs and a more satisfied work force [14–17].

Health economics offers an analytical technique to measure the financial impact of health-promoting initiatives in order to assess allocation efficiency and determine whether or not an intervention is worthwhile. Although it is important for government, organisations and businesses to accurately measure the rate of return on investments, the application of health economic theory in workplace health is steeped in methodological complexities [14]. Primarily, economic evaluations focus on indicators of business performance and health change targets. Although tools such as workplace health calculators are available for decision makers who wish to create a business case for workplace health, those that currently exist online have been developed from evidence arising out of the Unites States and the United Kingdom with financial estimates available in British pound [18, 19] and United States dollar [20], and the latter only suitable to businesses with greater than 1000 employees based in US, Europe, India and China. Little is available to assist other jurisdictions in the business case for workplace health, both in terms of currency output and simple translation, and as a result, the adoption of these existing online-calculators can be problematic.

In 2009 the Australian Government established the National Partnership Agreement on Preventive Health initially promising an investment of $221.8 million over nine years (2009–2010 to 2017–2018) [8]. This commitment provided funding to all states and territories to support the Healthy Workers Initiative and enabled Australian health policy-makers to engage in a common mission to improve and maintain the health and wellbeing of workers. With this support, a Healthy Workers Initiative project team was developed within Population Health Services in the Tasmanian Department of Health and Human Services. One of the many objectives of the project team was to develop an evidence-based, simple and easy-to-use resource (calculator) for Australian employers interested in workplace health investment figures, and make this available through the Healthy Workplace Resource Toolkit.

This paper describes the development of the Workplace Health Savings Calculator, a toolkit output that is currently available online.

## Data collection

Data were collected in three phases (1) locate appropriate effectiveness measures, (2) identify change estimates surrounding these measures and (3) decide on an appropriate model.

To satisfy the first phase, a literature review was being performed by SB, AP, KS and AV (the researchers) at the time the Healthy Worker Initiative project team members SC and CO approached with the question “What is the evidence-based business case for workplace health promotion?” A partnership agreement was established and researchers utilised their concurrent literature search for the purposes of providing economic evidence to assist the development of the Healthy Workplace Resource Toolkit. The search was conducted in relevant economic and biomedical databases between November 2011 and January 2012. In addition, a keyword search using Google Scholar and a manual search of citations from relevant papers was undertaken to locate published evidence on the financial impact of workplace health promotion. The search strategy has been published along with the review [14]. Information gained from this review was utilised to ascertain measures of effectiveness which contextually provided transferability and generalisability to the Australian sector. Two measures of effectiveness were recognised as business metrics most readily captured in operations. These were worker ‘absenteeism’ and ‘staff turnover’. Both were adopted as the key performance estimates for the calculator.

The second requirement in the development phase was to establish the magnitude of possible change in absenteeism and staff turnover as a result of implementing a workplace health program. These estimates of change for absenteeism and staff turnover were sourced from a second review study [21] which readers can refer to for additional information. This review, published in 2008, was commissioned by the Health Work Wellbeing Executive in England and undertaken by PriceWaterhouseCoopers LLP. Under the constraints identified in the first review, namely, that no Australian equivalent published data source existed, that volume of publications from the United States far exceeded that from jurisdictions operating under a national health care system, and that large variability in both estimates and methodological quality of studies prevail, the authors considered this PriceWaterhouseCoopers’ review to be most appropriate for our needs and of sound evidence base. Moreover, the evidence from this review is cited and supports the Workplace Wellbeing Charter [6], a national award, whose “standards reflect best practice” and is endorsed by Public Health England.

Finally, an internet search was conducted to locate workplace health calculators currently in existence. These were assessed for their ease of use and applicability to the Australian business context. As a result of this search, a model developed by the National Institute for Health and Clinical Excellence (NICE) [19] was considered simple to use and adapted for our purposes.

## Assumptions used to develop the tool

In developing the tool, the following assumptions were made. First, ‘absenteeism’ (or ‘sick leave’) was defined as an employee’s unplanned leave from work, not including other leave such as carer’s leave or maternity leave. Examples of unplanned leave would be due to illnesses such as colds and flu.

Second, a workplace health promotion program was considered ‘successful’ when it was designed to target the needs of employees, when participation rates were reasonable (greater than 25 % participation), and the program was actively supported by senior management and leaders within the organisation.

Third, different types of workplace health promotion interventions (health and safety, disease management, and health promotion—the modification of risk behaviours such as smoking, nutrition, physical activity and stress to improve overall employee wellbeing) contributed equally, and were linked to the improvement of the effectiveness estimates.

Last, calculated savings were assumed to be a long-term benefit. It is evidenced in the literature that positive effects on absenteeism and staff turnover occur between 2 and 5 years post implementation of a successful workplace health program [22].

The PriceWaterhouseCoopers’ review [21], from which the magnitude of change for absenteeism and staff turnover was sourced, included 55 case studies from organisations in the United Kingdom that implemented a variety of workplace health promotion programs. The case studies were submitted to the Health Work Wellbeing Executive and PricewaterhouseCoopers LLP was commissioned to undertake a review including interviews with selected organisations. Overall, 45 case studies reported evidence on change related to absenteeism and 18 on staff turnover, with 28 (51 %) providing evidence from behaviour modification or lifestyle programs such as smoking cessation, healthy diet and subsidised exercise programmes. These interventions focussed on similar behavioural and lifestyle health risk change targets to those encouraged in Australia, which are commonly referred to as SNAPS (smoking, nutrition, alcohol, physical activity, stress) interventions [23]. There were 32 case studies (58 %) focussed on occupational health and safety interventions. The data were collected from businesses within nine different industries; defined as manufacturing, finance, public service, utilities, business services, construction/engineering, retail, education, and others. Company size and intervention type by industry group for all case studies is provided in Appendix 2b of the source review [21]. Their diversity represented a good range of industry types relevant to Australia, with national statistics identifying the vast majority of Australian businesses operate in the service sectors (construction, professional/scientific/technical, retail trade, education, accommodation, transport, and utilities), with the remaining in manufacturing, mining agriculture/forestry and fishing [24]. Further similarities between these two nations such as the proportion of small to medium businesses, population demographics and drivers for workplace health promotion are shown in Table 1.

**Table 1:** Generalisability of the source review

Parameters	Australia	United Kingdom (UK)	Comments/assumptions
SME proportion	99.7 % [42]	99.9 % [43, 45]	UK effectiveness estimates in report derived from similarly high proportion of SMEs to Australia*
Industry types	85 % of SMEs operate in the service sectors (construction (14 %), professional/scientific/technical (12 %), retail trade (10 %) and others including education, accommodation, transport, utilities), with the remaining in agriculture/forestry and fishing (8 %), manufacturing (6 %) and mining (1 %) [24]	Data from 9 industries: manufacturing, finance, public service, utilities, business services, construction/engineering, retail, education, others [21]	Good range of industry types relevant to Australian industry. Construction industry reported effectiveness for occupational health and safety (OH&S) interventions only
Aging population	In 2005, median age 36.6 years [46] By 2050, median age 45 [47] 1 in 4 Australians aged 65 years or over by 2056 [48]	In 2005 median age 39 years [46] By 2050, median age 43 [47] Between 1971 and 2006, those aged 65 years increased by 31 % [21]	Similar population aging demographics
Aging workforce	By 2050, 26 % over 65 years [49]	By 2024, 50 % over 50 years [50] By 2050, 24 % over 65 years [49]	Similar workforce demographics
Drivers	Human capital**, government initiative, OH&S [51]	Government, social responsibility, rising cost of human capital [21]	Similar implementation drivers
Intervention targets	SNAPS (i.e.: smoking, nutrition, alcohol, physical activity, stress) behavioural and lifestyle health risks [23]	51 % (28/55) lifestyle (i.e.: smoking cessation, healthy diet and subsidised exercise programmes) 58 % (32/55) OH&S [21]	Lifestyle interventions focus on similar behaviour change targets to those encouraged in Australia and are also those most commonly seen in research of behaviour modification health interventions in the workplace

Source review: PricewaterhouseCoopers LLP was commissioned by the Health Work Wellbeing Executive to undertake a review of the business case for workplace health, which included a review of 55 case studies from United Kingdom organisations [21]

* There were seven SMEs (small to medium enterprises) of the 55 case studies in the source review; two measured absenteeism, one measured staff retention, three measured both absenteeism and staff retention, and one measured absenteeism (from OH&S interventions only). In their reported benefits, all SMEs saw decreased absenteeism and improved retention

** Human capital: drivers include talent attraction, retention and ideas of broader corporate social responsibility. This approach also seeks to improve productivity and reduce workforce absenteeism [51]

Global trends in employer wellbeing strategies and practices were reported in 2014 [25]. Data were collected from 37 countries (in 11 languages) that included 1041 employer-participants (8 million employees) across all industry categories. Although it documented similarities between Australia/New Zealand and Europe in terms of percentages of organisations offering health promotion, health risk drivers (namely stress, physical activity, nutrition), and types of program components, no evidence relating to differences in effect size between countries was obtained. There is paucity in the literature surrounding between-country magnitudes of effect in workplace health promotion. Consequently, within the calculator, functionality allows change estimates for absenteeism and staff turnover to be edited by the user, and the default figure represents the lowest effectiveness estimate from the range reported in the UK PricewaterhouseCoopers’ review. Refer to Table 2 for change estimates and ranges. This most conservative approach acknowledges that these benefits may not be fully transferable to the Australian context.

**Table 2:** Change estimates used within the Workplace Health Savings Calculator

Change estimate	Source	Measurement	Assumption
Absenteeism (% decrease)	PWC 2008 [21]^a^	Average 30–40 % reduction, based on 45/55 case studies	The other 10 studies did not measure the perceived benefits of AB, so average holds for all that do
Staff turnover (replacement cost)	ABS 2008 [26]	75–150 % salary as replacement cost Industry types: engineering, construction, professional services (e.g.: finance, admin), public service, resources (e.g.: agriculture, mining) retail and entertainment	75 % a conservative assumption used in place of conclusive evidence
Staff turnover (% decrease)	PWC 2008 [21]	10–25 % decrease in staff turnover, based on 18/55 case studies. On average this retention range was 20–25 % (from 4 industry categories: finance, utilities, business service, and other)	That 37 case studies did not report on turnover, average based on the 18 studies that did. Average holds as an average for all

^a^These were extracted from the source review [21] of 55 case studies that had varying durations of implementation. It has been shown in the literature that benefits from reduced absenteeism and staff turnover may not be realised before 2 and 5 years after implementation of a successful workplace health promotion program [22]. We wish to reiterate an assumption outlined in this study that the calculated potential annual savings is a long-term benefit

When an average effectiveness estimate was reported, it was assumed the average was an average across the case studies that measured that particular effectiveness outcome. It was therefore presumed the average would apply for any business that measured these particular outcomes after implementation of a workplace health promotion program.

In concluding the assumptions used to develop the Workplace Health Savings Calculator, this tool is considered by the authors to be most appropriate for use in Australia, on the following basis; (1) input estimates for absenteeism and staff turnover are generated by the Australian user company, (2) cost estimates are derived using Australian wage statistics, and (3) change estimates from the PriceWaterhouseCoopers’ review are (a) most conservative and (b) generalizable to the Australian business context. The Workplace Health Savings Calculator specifically does not attempt to measure or quantify in dollar value any additional health benefits that may be enjoyed by employees undertaking health promotion in their workplace; as such estimates remain elusive in the literature [14].

## Description of user interface

**Fig. 1:**
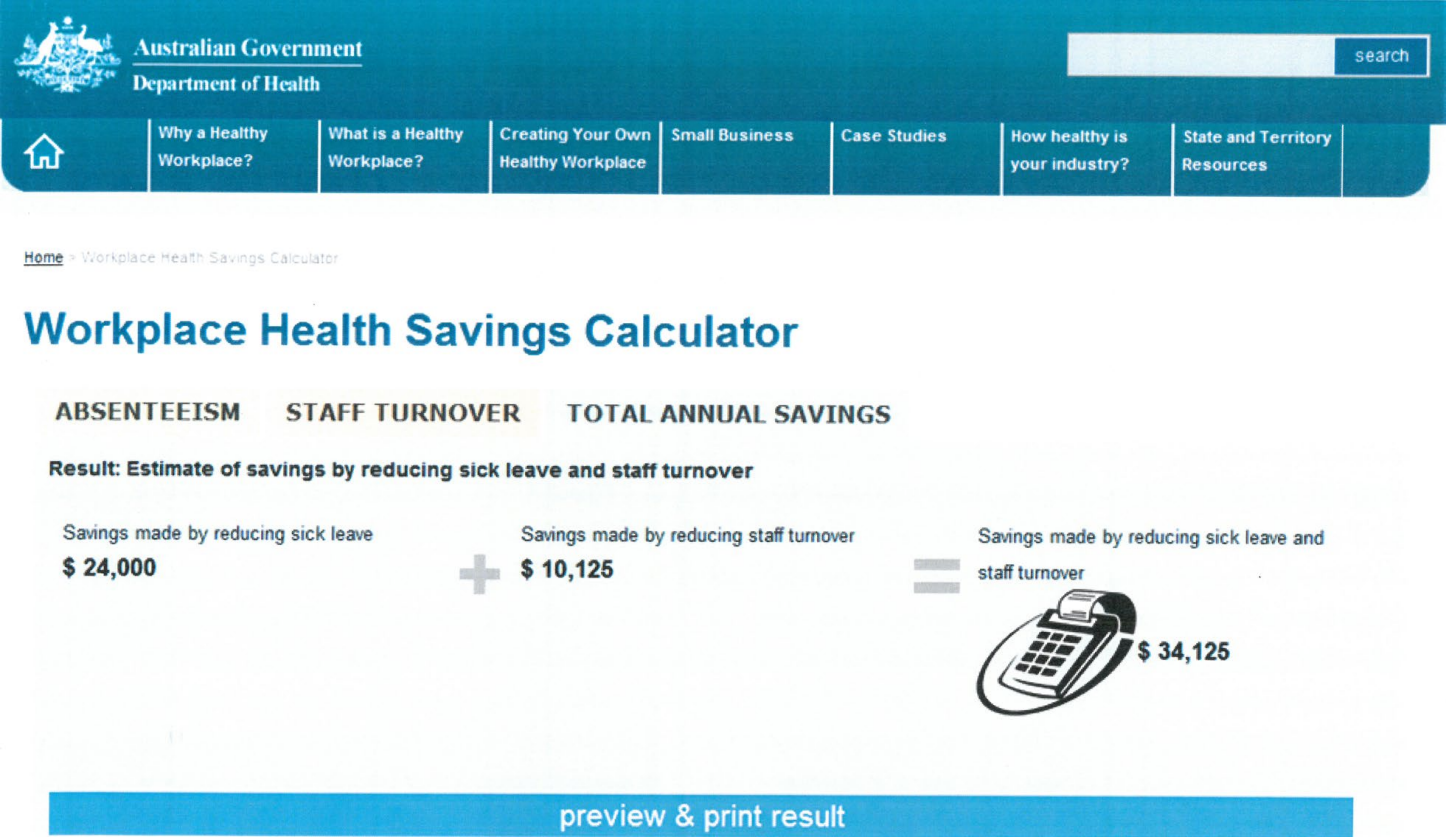
Workplace Health Savings Calculator as it appears on the Commonwealth Government’s Department of Health, Healthy Workers web portal. The following scenario is an example of a company profile whose input would match these calculations. In the last 12 months, a company of 100 employees has experienced a sick leave rate of 4 days per employee (total annual sick days 400) and has recruited 3 replacement staff. The average staff salary is $45,000. The company operates 8 h a day and the average hourly wage is $25. The estimated potential savings to the company when implementing a successful workplace health and wellbeing program is set at the default effectiveness measures; a 30 % reduction in sick leave and a 10 % reduction in staff turnover. The cost of replacing an employee is defaulted at 75 % of the annual salary

**Fig. 2:**
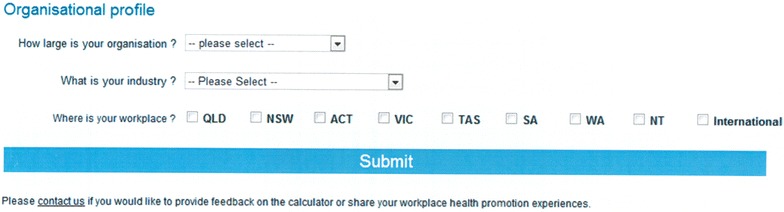
Screen that accompanies the Workplace Health Savings Calculator for purposes of data collection. The data is non-identifiable unless users wish to identify themselves by submitting an email via the ‘Contact us’ hyperlink option at the bottom of this organisational profile box

The calculator was adapted from a model developed by the National Institute for Health and Clinical Excellence (NICE) [19], and consists of three tabs (Fig. 1). The first allows the user to input relevant data on employee numbers and salary, the second to input data on staff turnover, and the third tab calculates the total potential annual savings that arise from the implementation of a successful workplace health promotion program. Below the savings output on this third and final tab is an organisational profile box which users have the option to complete and submit (Fig. 2). The submitting user maintains anonymity of the company name yet provides the site administrator with base level information about the company, such as industry type, business size and locality. Lastly, for users who wish to identify themselves, there is an option at the bottom of the box to submit an email via a ‘Contact us’ hyperlink.

For companies whose staffing profile does not solely consist of full-time employees, an additional feature was added to account for part-time and casual positions. For these businesses, where total number of full-time equivalent hours may not be recorded, there is an option within the calculator that allows the user to input ‘total number of sick days in the last 12 months’ instead of ‘total number of employees’. This feature simplifies the data gathering process, and allows users to choose between two algorithms in order to estimate, with minimal burden, the total annual savings in sick leave achievable by implementing a successful workplace health and wellbeing program.

Tabs one and two use effectiveness estimates to derive savings that arise from reduced absenteeism and staff turnover, which is defaulted to the most conservative estimates and can be overridden by the user. It was envisioned that the default estimates may be overridden by companies that are already implementing a program for which company-specific evaluation data were available, and for whom an online-generated calculation of annual savings offered some utility.

The effectiveness estimates within the calculator are sourced from the PriceWaterhouseCoopers’ review [21] and Australian wage statistics [26]. These were absenteeism rates, which reduce by an average of 30–40 % [21]; staff turnover rates, which decrease by 10–25 % [21]; and replacement cost due to staff turnover, which ranged from 75 to 150 % of the worker’s wage, an Australian national estimate [26]. There were many and various costs associated with this measure, such as costs for recruitment, training, specialist knowledge and productivity [27] which could account for the large range that was reported. In line with agreed assumptions, the most conservative estimates were used in the model when a range of estimates were offered. Details of these change estimates used and generalisability are provided in Tables 1 and 2.

The calculator was initially published in print within the Healthy Workplace Resource Toolkit (Table 3) with an accompanying page offering an example of the algorithm (Table 4). In 2013 a Microsoft Excel spreadsheet was developed and the calculator was published on the WorkSafe Tasmania website [28].

**Table 3:**
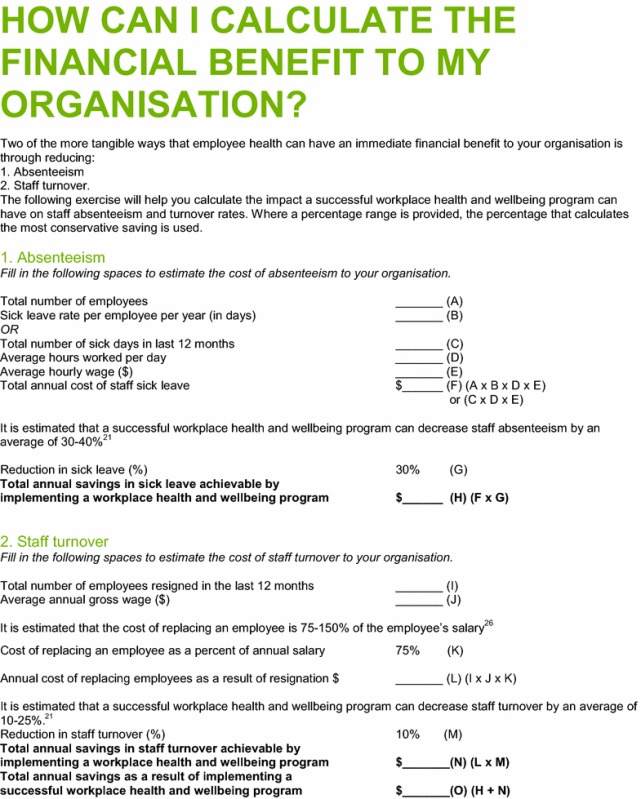
Print version of the simple Workplace Health Savings Calculator as it appeared in the Healthy Workplace Resource Toolkit

**Table 4:**
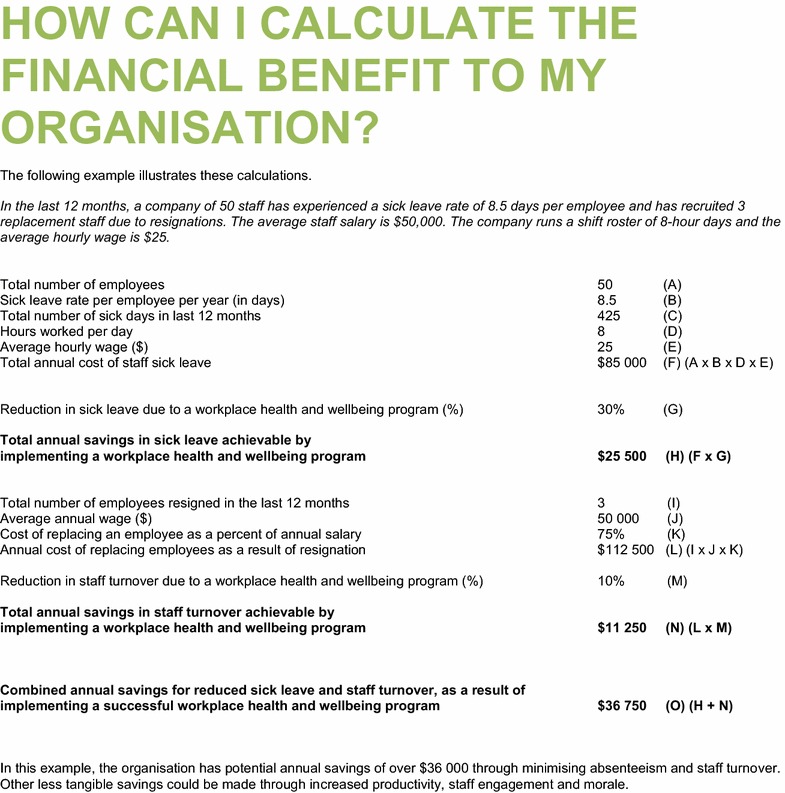
Example which accompanied the simple Workplace Health Savings Calculator in the Healthy Workplace Resource Toolkit

The algorithm was later adapted and reproduced by the Australian Government Department of Health and Ageing for use on the Healthy Workers web portal, as part of its official toolbox for the economic assessment of workplace health promotion programs. Titled “The Workplace Health Savings Calculator”, it is available at: http://www.healthyworkers.gov.au on the home screen in the ‘News’ link (or via direct link: http://www.healthyworkers.gov.au/internet/hwi/publishing.nsf/Content/roi-introduction).

Since its national online publication, the tool has been endorsement by an Australian non-government organisation and commercial providers of workplace health promotion and their respective networks. Further adaptions of the calculator can be viewed online [29, 30]. Evidence regarding its usability and further application are being collected through the organisational profile box and ongoing collaborator consultations. Initial data from the first year demonstrate the calculator has been accessed by a variety of businesses within the industries of Agriculture, Forestry & Fishing; Health and Community Services; Education; Government Administration and Defence; Retail; Electricity, Gas and Water; and Personal and Other Services. Data also indicate these businesses are located across every state and territory in Australia, and in both metropolitan and regional areas. Two international companies have also completed the organisational profile. The majority of organisations (88 %) employed less than 200 workers of which 40 % identified as small in size (1–19 employees). These initial statistics are encouraging, and not only demonstrate an interest in workplace health promotion from the Australian small-to-medium enterprise (SME) community but also across the entire country.

## Discussion

The Workplace Health Savings Calculator is an online tool for estimating the economic impact of improved productivity from the implementation of a successful workplace health promotion program. It utilises a conservative set of assumptions to generate an estimate of potential annual savings. It calculates financial benefits related to reduced absenteeism and staff turnover using input estimates (number of employees, sick leave rates, average hours worked, average wage, number of resignations) that are generated at the individual company level. Annual turnover and number of employees are tangible key performance estimates most commonly measured in Australia [24]. The estimate for cost to replace staff is an Australian statistic [26]. Although commonly measured, there is a lack of Australian evidence on absenteeism and staff turnover in relation to workplace health promotion outcomes and the authors were required to carefully consider the vast and varying evidence on effectiveness and cost-effectiveness in the global literature. This was achieved in concurrence with a systematic review undertaken by the authors SB, AP, KS and AV [14]. It was considered that these two metrics (absenteeism and staff turnover) provided (1) the ease of measurement needed, and (2) best attainable estimates to attribute a dollar value, and thereby met our primary objective to develop an evidence-based, simple and easy-to-use resource (calculator) for Australian employers interested in workplace health investment figures.

Presenteeism, being present at work while suffering from a health problem that may limit job performance [31], is also linked with negative impacts to productivity and associated costs. Indeed, presenteeism accounts for greater aggregate productivity loss than absenteeism [32–34], thus decreasing worker presenteeism rates will lead to greater savings. Although preliminary evidence has shown that workplace health promotion may be effective at decreasing presenteeism rates [35], there are critical issues surrounding the measurement, conversion and translation of value into economic outcomes [36–38]. It is not the intention of this calculator to overestimate outcomes or in the interest of sustainability of engagement for users to receive an inflated savings figure which may not be realised. For this reason, only business estimates from absenteeism and staff turnover were considered and the most conservative estimates were utilised when average ranges were reported.

The authors further acknowledge that estimating economic savings from productivity loss, even with the exclusion of a measure for presenteeism, remains debatable due to the wide variability, large influence on saving outputs, and issues surrounding use of indirect costs such as double counting and perspective [39]. Therefore the computed savings estimate from the Workplace Health Savings Calculator should not be considered to have utility in a health economic evaluation of workplace health promotion program. It is not an assessment or evaluation tool, rather an engagement tool to support workplace health and wellbeing efforts. The intended design and application is to engage businesses who are seeking an instrument to develop commitment at a stakeholder level.

Furthermore, the Workplace Health Savings Calculator is not a return on investment tool. It does not give the option to quantify program costs and therefore does not estimate net benefits or utilise cost benefit analysis techniques.

The United Kingdom PriceWaterhouseCoopers’ review [21] was considered to have a strong methodological approach for the reported business outcomes, with its published effectiveness data also being used to support the Workplace Wellbeing Charter, National Award for England. The authors believe this review represented the best evidence base. In a field known to be lacking in robust quantifiable effectiveness and economic data, the authors recognise the lack of a more scientific approach compromises the validity of the calculator however consider the findings from the case studies to be real world representation and their use in this tool a pragmatic application.

Moreover, the NICE model from where the Workplace Health Savings Calculator was adapted is available as a business case tool within the NICE guidelines [PH13] for promoting physical activity at work. In December 2014 the guidelines underwent a second three-yearly review and the concluding decision states “no new evidence was identified which appeared to contradict the existing recommendations” [40]. Reliability and validity are cornerstone principles to scientific method, and although a gross limitation to the calculator is the fact that neither has been tested, the continued and ongoing expert opinion accepts such limitations due in part to a lack of rigorous evaluation designs, and the complexities and heterogeneities surrounding this public health intervention.

In terms of generalisability, the research evidence used for change estimates was generated from an international (UK) context not an Australian setting where the calculator is applied. It is therefore unknown whether the effect size is transferable to locally-implemented interventions. However, we demonstrated that business sector statistics, workplace health strategies and practices, and the overarching political agenda focused on promoting health in the workplace to address rising prevalence of chronic disease is similar between both countries. Baseline prevalence, characteristics of the target population and capacity to implement interventions are key attributes for transferability in evidence-based public health [41].

From the initial data on organisational profile collected by the online Workplace Health Savings Calculator there has been a large proportion of SME interest. Australia defines a SME as a business employing 0–199 workers (small represents 0–19 employees and medium represents 20–199 employees [24]), and SMEs make up 99.7 % of the Australian business sector [42]. This is comparable in both proportion and definition to United Kingdom, where SMEs are “businesses with zero to 249 employees, (which) account for 99.9 per cent of all enterprises” [43]. Interestingly, of the 55 case studies in the source review, only seven (13 %) were SMEs, representing manufacturing, financial, business services and retail sectors. The approximate size for all other organisations ranged from 200 to 100,000+, the largest being the public sector service organisation. The low representation by small-to-medium business in the review could indicate a general lack of engagement or lack of resources. Nevertheless, in jurisdictions and regions where the business profile differs, for example in Tasmania, Australia (where the vast majority of SMEs are small businesses (94.8 %), with 58.8 % being non-employing businesses and 36 % employing 0–19 workers [42, 44]), a declaration of company size from where estimates originated should be made within the calculator.

Workplace health promotion is a modern corporate strategy, and for countries like Australia, it is a recognised public health initiative aimed at improving employee health and wellbeing. Calculators to assist in business justification are needed to develop stakeholder commitment and are seen as suitable to engage business in conversation for promoting health in the workplace. Other currently available online calculators lack generalisability to the Australian business market. Limitations surround country specificity, currency, complexity and appropriate evidence transferability. In contrast, the Workplace Health Savings Calculator is a practical easy-to-use business case tool that was developed in line with one of the core principles of the National Partnership Agreement on Preventive Health, and is to be used to support, engage and promote the implementation of healthy lifestyle programs in Australian workplaces.

## Availability and requirements

Project name: Workplace Health Savings Calculator

Project home page: http://www.healthyworkers.gov.au and direct link available at: http://www.healthyworkers.gov.au/internet/hwi/publishing.nsf/Content/roi-introduction

Operating system(s): Platform independent

Programming language: HTML

Other requirements: Nil

Any restrictions to use by non-academics: None (free to access).

### Availability of supporting data

The data supporting the results of this article are included within the article and its additional files.

## Abbreviations

SME: Small to medium enterprise
WHO: World Health Organisation
UK: United Kingdom
NICE: National Institute for Health and Clinical Excellence
